# Pregnancy intention and contraceptive use among HIV-positive Malawian women at 4-26 weeks post-partum: A nested cross-sectional study

**DOI:** 10.1371/journal.pone.0215947

**Published:** 2019-04-23

**Authors:** Deus Thindwa, Megan Landes, Monique van Lettow, Annie Kanyemba, Ernest Nkhoma, Happy Phiri, Thokozani Kalua, Joep J. van Oosterhout, Evelyn J. Kim, Beth A. Tippett Barr, on behalf of the NEMAPP Consortium

**Affiliations:** 1 Management Sciences for Health, Lilongwe, Malawi; 2 Dignitas International, Zomba, Malawi; 3 Department of Family and Community Medicine, University of Toronto, Ontario, Canada; 4 Dalla Lana School of Public Health, University of Toronto, Ontario, Canada; 5 Department of HIV, Ministry of Health, Lilongwe, Malawi; 6 Department of Medicine, College of Medicine, University of Malawi, Blantyre, Malawi; 7 Centers for Disease Control and Prevention, Lilongwe, Malawi; Boston University School of Public Health, UNITED STATES

## Abstract

**Background:**

Avoiding unintended pregnancies through family planning is a WHO strategy for preventing mother to child transmission of HIV (PMTCT) and maternal morbidity/mortality. We investigated factors associated with unintended index pregnancy, unmet contraceptive need, future pregnancy intention and current contraceptive use among Malawian women living with HIV in the Option B+ era.

**Methods:**

Women who tested HIV positive at 4–26 weeks postpartum were enrolled into a cross-sectional study at high-volume Under-5 clinics. Structured baseline interviews included questions on socio-demographics, HIV knowledge, partner's HIV status/disclosure, ART use, pregnancy intention and contraceptive use. Logistic regression was used to determine factors associated with outcomes.

**Results:**

We enrolled 578 HIV-positive women between May 2015-May 2016; median maternal age was 28 years (y) (interquartile-range [IQR]: 23–32), median parity was 3 deliveries (IQR: 2–4) and median infant age was 7 weeks (IQR: 6–12). Overall, 41.8% women reported unintended index pregnancy, of whom 35.0% reported unmet contraceptive need and 65.0% contraceptive failure. In multivariable analysis, unintended index pregnancy was higher in ≥35y vs. 14-24y (adjusted Odds Ratio [aOR]: 2.1, 95% Confidence Interval [95%CI]: 1.0–4.2) and in women with parity ≥3 vs. primiparous (aOR: 2.9, 95%CI: 1.5–5.6). Unmet contraceptive need at conception was higher in 14-24y vs. ≥35y (aOR: 4.2, 95%CI: 1.8–9.9), primiparous vs. ≥3 (aOR: 8.3, 95%CI: 1.8–39.5), and women with a partner of unknown HIV-status (aOR: 2.2, 95%CI: 1.2–4.0). Current contraceptive use was associated with being on ART in previous pregnancy (aOR: 2.5, 95%CI: 1.5–3.9).

**Conclusions:**

High prevalence of unintended index pregnancy and unmet contraceptive need among HIV-positive women highlight the need for improved access to contraceptives. To help achieve reproductive goals and elimination of MTCT of HIV, integration of family planning into HIV care should be strengthened to ensure women have timely access to a wide range of family planning methods with low failure risk.

## Background

By 2017, 36.9 million people worldwide were living with human immunodeficiency virus (HIV), the majority (53.1%) in sub-Saharan Africa [1]. Malawi has one of the highest adult HIV prevalence at 9.3% [2,3], and women of reproductive age comprise 53.5% of all 15–49 year olds living with HIV in Malawi [4]. In July 2011, Malawi adopted a test-and-treat strategy for the prevention of mother to child transmission of HIV (PMTCT) for HIV-positive pregnant and breastfeeding women, known as Option B+. Early evaluations of Option B+ have shown markedly increased uptake of ART among pregnant and breastfeeding women in Malawi [5], with current PMTCT coverage estimated at 93.5% among pregnant women and early vertical HIV transmission reduced to an estimated 3.9% [6]. However, more work is required to achieve virtual elimination of mother-to-child transmission of HIV, defined as having ≤50 new paediatric infections per 100,000 live births and a transmission of either <5.0% in breastfeeding populations or <2.0% in non-breastfeeding populations [7].

The World Health Organization (WHO) has listed preventing unintended pregnancies in HIV-positive women as a key PMTCT strategy [8,9]. A mathematical modelling study based on data from Uganda predicted that the effect of preventing unintended pregnancies in HIV-positive women could contribute to PMTCT equally or more than ART [10]. Family planning and contraceptive use have demonstrated to be cost-effective interventions for PMTCT and preventing maternal morbidity and mortality from unintended pregnancies [11]. To improve access to and uptake of family planning and contraceptives among women living with HIV, the integration of family planning into HIV care and treatment services has been proposed. Pilot studies in Malawi, Kenya, Tanzania, Swaziland, Nigeria, South Africa, and Uganda have shown that this is acceptable, feasible and cost-effective [12–16]. In 2011, the Malawi HIV programme integrated family planning into HIV care and treatment services in all ART facilities [17,18]. However, the integrated system is challenged by insufficient policy enforcement, infrastructure, funding and leadership [18].

Despite these advances, prevalence of unintended pregnancies and unmet contraceptive need remain high among HIV-positive women in sub-Saharan African and particularly in Malawi [19–22]. There is evidence that pregnancy intention among women in Malawi decreases after HIV seroconversion [8,19,23]. Determinants of pregnancy intention and contraceptive use among women at risk of HIV infection in Malawi include age, employment, marital and education statuses, knowledge about ovulatory cycle and number of living children [4,24], but knowledge of determinants among HIV-positive women is still limited, and we know of no literature on these topics from Option B+ programmes globally. We describe index and future pregnancy intention and contraceptive use among women who were 4–26 weeks post-partum within the Malawi National PMTCT program. Additionally, we describe factors associated with unintended index pregnancy and unmet contraceptive need, as well as with future pregnancy intention and current contraceptive use.

## Methods

### Study design and participants

This is a cross-sectional study nested within a nationally representative cohort of HIV-positive Malawian women who were enrolled at 4–26 weeks post-partum in the National Evaluation of the Malawi PMTCT Programme (NEMAPP) study. The parent NEMAPP study used a multistage cluster design to randomly select 54 health facilities across Malawi where mother-infant pairs were consecutively consented, interviewed, and screened for HIV; women testing HIV-positive were invited to participate in the cohort study. In this cross sectional sub-study, we enrolled HIV-positive women presenting with their 4-26-week-old exposed infants at the Under-5 clinics of three government health facilities (1 hospital and 2 health centres) that were purposefully selected because of their large patient volumes and representation of urban and rural settings. Women were interviewed at enrolment on socio-demographics, clinical characteristics, index and future pregnancy intentions and contraceptive use by trained health facility staff using structured questionnaires (S1 Appendix).

### Study outcome definitions

We defined unintended index pregnancy as the pregnancy of a present 4–26 weeks old infant, which was unwanted or mistimed at the time of conception. Unmet contraceptive need was defined as the proportion of women whose index pregnancy was unintended, but did not report contraceptive use at the time of conception. Future pregnancy intention was defined as the desire to have another child within or after 12 months from the time of enrolment. Current contraceptive use was defined as the proportion of women who were fertile, sexually active and were using a method of contraception to stop child bearing or delay pregnancy for the next 12 months.

### Data collection and statistical analysis

We designed standardized questionnaires to record socio-demographics, clinical characteristics and family planning-related outcomes of study participants. Exposure variables included age, parity, previous child death, education level, religion, HIV test in index pregnancy, HIV result if tested in index pregnancy, disclosure of HIV status to partner, known HIV status of partner, partner’s HIV result if known, ART in previous pregnancy, timing of ART, health status at ART initiation, and health status at enrolment. Data were pooled across the three sites and the site population effects were not modelled. Our sample also included a small group of women who tested positive during enrolment but reported HIV-negative during index pregnancy, suggesting that they seroconverted later during pregnancy or post-partum. Descriptive statistics were used to characterise study participants and estimate the proportion of each outcome, stratified by socio-demographic and clinical characteristics. Exposure variables which had statistically significant 95% confidence intervals (95%CI) Mantel-Haenszel crude odds ratio for at least one comparison group were considered as potential factors for association with study outcome. We included each potential factor in multivariate logistic models using a step-wise forward algorithm at the significance level of a Likelihood Ratio Test, *P* <0.05 [25]. Final multivariate logistic models controlled for confounders and risk factors which included age, parity, education level, religion, timing of ART, known HIV status of partner, partner’s HIV result if known, HIV result if tested in index pregnancy, ART in previous pregnancy, health status at ART initiation and future pregnancy intention. Any two given variables were also checked for collinearity, and one variable was omitted if its variance inflation factor was greater than five. Analyses were carried out using Stata version 14.0 (StataCorp, College Station, TX, USA).

### Ethical considerations

Ethical approval was obtained from the Malawi National Health Sciences Research Committee (NHSRC, #1262), the Centers for Disease Control and Prevention Center for Global Health Associate Director for Science (#2014-054-7) and the University of Toronto Research Ethics Committee (#30448). Participants provided written informed consent to enrol in the study.

## Results

### Descriptive characteristics

Five hundred and seventy-eight women were enrolled in the study between May 2015 and May 2016. The median maternal age was 28 years (n = 576, interquartile range [IQR]: 23–32), median parity was 3 deliveries (n = 578, IQR: 2–4) and median infant age was 7 weeks (n = 578, IQR: 6–12). Overall, 240 women (41.8%) reported the index pregnancy as unintended, among whom 84 (35.0%) reported unmet contraceptive need. Of the remaining 156 women (65.0%) who did not intend their index pregnancy but who were using contraceptives at the time of conception, 100 (64.1%) reported using depot-medroxy-progesterone acetate, 23 (14.7%) used depot-levonorgestrel, 16 (10.3%) always used condoms, 13 (8.4%) took oral contraceptives, 1 (0.6%) had an intrauterine contraceptive device (IUCD) and 3 (1.9%) used other methods. Among 109 women who reported unintended index pregnancy and used contraceptives, only 9/84 (10.7%) and 4/25 (16.0%) reported partners’ HIV positive and negative status respectively, and used condoms.

Of 470/528 women (89.0%) who reported knowing their positive HIV result during index pregnancy, 198/470 (42.1%) had an unintended index pregnancy, of whom 70/198 (35.4%) had an unmet contraceptive need. Pregnancy intention and contraceptive need of the other 58 of 528 (11.0%) women who recently seroconverted did not significantly differ from early seroconverters (69.0% vs. 57.9%; p = 0.105) and (51.7% vs. 60.0%; p = 0.226) respectively. A total of 361/548 (65.9%) women knew their partner’s HIV status, of whom 99/361 (27.4%) said the partner was HIV-negative. One hundred and seventeen of 260 (45.0%) women who knew their partner was HIV-positive and who stated their index pregnancy was unintended, 33/117 (28.2%) were not using contraception. Women in higher age categories, in higher parity categories, who had started ART before the index pregnancy, whose partner had HIV-positive result, or who had used contraceptives before the index pregnancy had significantly higher prevalence of unintended index pregnancy. Women in higher age categories, in higher parity categories, who disclosed their HIV status to partner, who had used a triple drug ART regimen in previous pregnancy or had unintended index pregnancy had significantly higher prevalence of contraceptive use before the index pregnancy (Table 1).

**Table 1:** Maternal characteristics by pregnancy intention and contraception use related to the index pregnancy as reported at 4–26 weeks post-partum.

	Overall	Index pregnancy intention**			Contraception use**		
	(N = 578)	(N = 574)			(N = 576)		
Infant median age [IQR]: (7 [6–12])		Intended	Unintended	*Difference*^¶^	Yes	No	*Difference*^¶^
Maternal median age [IQR]: (28 [23–32])	N (%)	n (%)	n (%)	% (95%CI)	n (%)	n (%)	% (95%CI)
**Total**		334	240		339	237	
**Age group (years)**							
14–24	176 (30.6)	120 (36.0)	55 (23.0)	13.0 (5.1–20.5)^#^	78 (23.1)	97 (41.1)	-18.0 (10.0–25.9)^#^
25–34	314 (54.5)	189 (56.8)	124 (51.9)	4.9 (-3.6–13.3)	201 (59.5)	113 (47.9)	11.6 (3.1–19.9)^#^
≥ 35	86 (14.9)	24 (7.2)	60 (25.1)	-17.9 (11.6–24.5)^#^	59 (17.4)	26 (11.0)	6.4 (0.3–12.2)^#^
**Parity**							
Median parity [IQR]	[3 (2–4)]						
One	92 (15.9)	69 (20.7)	23 (9.6)	11.1 (4.9–16.9)^#^	11 (3.2)	81 (34.2)	-31.0 (24.4–37.6)^#^
Two	148 (25.6)	103 (30.8)	43 (17.9)	12.9 (5.5–19.9)^#^	94 (27.7)	52 (21.9)	5.8 (-1.7–13.0)
Three or more	338 (58.5)	162 (48.5)	174 (72.5)	-24.0 (15.7–31.7)^#^	234 (69.0)	104 (43.9)	25.2 (16.7–33.1)^#^
**Previous child death**^Ɨ^							
None	382 (78.6)	210 (79.2)	169 (77.9)	1.3 (-6.2–9.1)	261 (79.6)	119 (76.3)	3.3 (-4.6–12.0)
At least one	104 (21.4)	55 (20.8)	48 (22.1)	-1.3 (-6.2–9.1)	67 (20.4)	37 (23.7)	-3.3 (-4.6–12.0)
**Education level**							
None	55 (9.6)	33 (9.9)	21 (8.8)	1.1 (-4.3–6.5)	32 (9.4)	23 (9.7)	-0.3 (-4.7–5.8)
Primary	314 (54.5)	178 (53.3)	135 (56.5)	-3.2 (-5.3–11.6)	189 (55.8)	125 (53.0)	2.8 (-5.7–11.3)
At least secondary	207 (35.9)	123 (36.8)	83 (34.7)	2.1 (-6.2–10.2)	118 (34.8)	88 (37.3)	-2.5 (-5.7–10.7)
**Religion**							
Christianity	496 (87.0)	284 (86.3)	208 (87.8)	-1.5 (-4.6–7.2)	296 (87.8)	198 (85.7)	2.1 (-3.9–8.3)
Islam	44 (7.7)	27 (8.2)	17 (7.2)	1.0 (-4.0–5.6)	23 (6.8)	21 (9.1)	-2.3 (-2.4–7.4)
Other religion	30 (5.3)	18 (5.5)	12 (5.0)	0.5 (-4.0–4.3)	18 (5.4)	12 (5.2)	0.2 (-4.3–4.1)
**HIV test in index pregnancy**							
No, already known HIV positive	42 (7.4)	20 (6.0)	20 (8.5)	-2.5 (-2.1–7.5)	24 (7.1)	18 (7.7)	-0.6 (-4.0–5.6)
Yes, tested for HIV	529 (92.6)	312 (94.0)	216 (91.5)	2.5 (-2.1–7.5)	312 (92.9)	216 (92.3)	0.6 (-4.0–5.6)
**HIV result if tested in index pregnancy**							
Negative	58 (11.0)	40 (12.8)	18 (8.3)	4.5 (-1.3–9.9)	30 (9.6)	28 (13.0)	-3.4 (-2.3–9.5)
Positive	471 (89.0)	272 (87.2)	198 (91.7)	-4.5 (-1.3–9.9)	282 (90.4)	188 (87.0)	3.4 (-2.3–9.5)
Disclosure of HIV status to partner^*^							
Yes	429 (81.6)	255 (82.3)	174 (80.9)	1.4 (-5.5–8.6)	262 (84.8)	167 (77.3)	7.5 (0.5–14.8)^#^
No	67 (12.7)	39 (12.6)	28 (13.0)	-0.4 (-5.5–6.8)	36 (11.7)	31 (14.4)	-2.7 (-3.3–9.1)
No partner	30 (5.7)	16 (5.1)	13 (6.1)	-1.0 (-3.3–5.7)	11 (3.5)	18 (8.3)	-4.8 (0.5–9.8)^#^
**Known HIV status of partner**^§1^							
HIV status unknown	187 (34.1)	108 (34.0)	78 (34.4)	-0.4 (-7.8–8.8)	103 (31.5)	83 (37.7)	-6.2 (-2.1–14.6)
HIV status known	361 (65.9)	210 (66.0)	149 (65.6)	0.4 (-7.8–8.8)	224 (68.5)	137 (62.3)	6.2 (-2.1–14.6)
**Partner’s HIV result if known**^§2^							
HIV negative	99 (27.4)	67 (31.9)	32 (21.5)	10.4 (0.6–19.6)^#^	61 (27.2)	38 (27.7)	-0.5 (-9.1–10.6)
HIV positive	262 (72.6)	143 (68.1)	117 (78.5)	-10.4 (0.6–19.6)^#^	163 (72.8)	99 (72.3)	0.5 (-9.1–10.6)
**ART in previous pregnancy**^Ŧ1^							
No ART in pregnancy	255 (53.4)	145 (55.4)	108 (50.9)	4.5 (-4.9–13.6)	162 (50.3)	93 (60.4)	-10.1 (0.2–19.6)^#^
sdNVP in pregnancy	57 (11.9)	31 (11.8)	26 (12.3)	-0.5 (-5.7–6.9)	35 (10.9)	22 (14.3)	-3.4 (-3.0–10.9)
ART in pregnancy	166 (34.7)	86 (32.8)	78 (36.8)	-4.0 (-4.9–12.9)	125 (38.8)	39 (25.3)	13.5 (4.1–22.1)^#^
**Timing of ART**^Ŧ2^							
Start before pregnancy of index baby	250 (43.9)	123 (37.4)	126 (53.4)	-16.0 (7.4–24.3)^#^	158 (47.6)	92 (39.1)	8.5 (-0.1–16.8)
Start during pregnancy of index baby	258 (45.4)	167 (50.8)	89 (37.7)	13.1 (4.5–21.3)^#^	143 (43.1)	114 (48.5)	-5.4 (-3.1–13.9)
Never on ART	61 (10.7)	39 (11.8)	21 (8.9)	2.9 (-2.6–8.2)	31 (9.3)	29 (12.4)	-3.1 (-2.4–8.8)
**Health status at ART initiation**							
No illness	407 (80.3)	242 (83.4)	162 (75.7)	7.7 (0.4–15.3)^#^	248 (82.1)	158 (77.5)	4.6 (-2.6–12.3)
A little bit sick	79 (15.6)	39 (13.5)	40 (18.7)	-5.2 (-1.5–12.3)	42 (13.9)	37 (18.1)	-4.2 (-2.5–11.3)
Very sick	21 (4.1)	9 (3.1)	12 (5.6)	-2.5 (-1.4–7.0)	12 (4.0)	9 (4.4)	-0.4 (-3.4–4.9)
**Health status at enrolment**							
No illness	551 (95.7)	316 (95.2)	231 (96.3)	-1.1 (-2.9–4.7)	325 (95.9)	224 (95.3)	0.6 (-3.1–4.7)
A little bit sick	25 (4.3)	16 (4.8)	9 (3.7)	1.1 (-2.9–4.7)	14 (4.1)	11 (4.7)	-0.6 (-3.1–4.7)
Very sick	0 (0.0)	0 (0.0)	0 (0.0)		0 (0.0)	0 (0.0)	
**Index pregnancy intention**							
Intended	334 (58.2)				183 (54.0)	151 (64.3)	-10.3 (1.8–18.4)^#^
unintended	240 (41.8)				156 (46.0)	84 (35.7)	10.3 (1.8–18.4)^#^
**Contraceptive use related to index pregnancy**							
Yes	339 (58.9)	183 (54.8)	156 (65.0)	-10.2 (1.8–18.3)^#^			
No	237 (41.1)	151 (45.2)	84 (35.0)	10.2 (1.8–18.3)^#^			

* Disclosure of HIV status to partner for only those with a known HIV result and with a partner. Chi-square *P* value excludes those in “No partner” category.

Ɨ Previous child deaths for those with at least two children.

Ŧ^1^ ART in previous pregnancies other than pregnancy of index baby. ^2^Timing of ART if they have been on ART ever since.

§^1^ Known HIV status of partner and ^2^partner HIV result if known ever since they became partners.

¶ Difference between proportions: pregnancy intention (intended vs. unintended) or contraceptive use(yes vs. no), and 95% Confidence Intervals (CI) with continuity correction.

# The difference between two proportions is statistically significant.

** Missing outcome data: index pregnancy intention (4), and contraceptive use (2). Missing categorical variable data were excluded in analysis.

ART–Antiretroviral therapy, HIV–Human Immunodeficiency Virus, sdNVP–single dose Neverapine.

NOTE: all percentages were rounded off such that they sum to 100% in each column for each factor.

A total of 136/529 women (25.7%) reported wanting a future pregnancy. Of 393/529 women (74.3%) who did not desire any future pregnancy, 195/393 (49.6%) were not currently using any contraceptive method. Additionally, of those wanting a future pregnancy, 125/136 women (91.9%) desired pregnancy after 12 months, and 73/125 (58.4%) of these women were not currently using any contraceptive method. Women in lower age categories, in primiparous or secundiparous categories, with at least secondary education, in other religion than Christianity, who started ART during the index pregnancy, or who were not currently using contraceptives had significantly higher prevalence of future pregnancy intention. Women with primary level education, who tested HIV-negative in index pregnancy, who had used a triple drug ART regimen, who had started ART before the index pregnancy, who were a little bit sick at ART initiation, or didn’t want future pregnancy had significantly higher prevalence of current contraceptive use (Table 2).

**Table 2:** Maternal characteristics by future pregnancy intentions and current contraception use as reported at 4–26 weeks post-partum.

	Overall	Future pregnancy intentions**			Current contraception use**		
	(N = 578)	(N = 529)			(N = 558)		
		Yes	No	*Difference*^¶^	Yes	No	*Difference*^¶^
	N (%)	n (%)	n (%)	% (95%CI)	n (%)	n (%)	% (95%CI)
**Total**		136	393		262	296	
**Age group (years)**							
14–24	176 (30.6)	73 (53.7)	90 (23.0)	30.7 (20.8–40.1)^#^	73 (28.1)	96 (32.4)	-4.3 (-3.6–12.1)
25–34	314 (54.5)	58 (42.6)	232 (59.3)	-16.7 (6.6–26.4)^#^	147 (56.5)	157 (53.0)	3.4 (-5.0–11.1)
≥ 35	86 (14.9)	5 (3.7)	69 (17.7)	-14.0 (7.7–18.8)^#^	40 (15.4)	43 (14.5)	0.9 (-5.3–7.2)
**Parity**							
One	92 (15.9)	52 (38.2)	34 (8.7)	29.5 (20.8–38.7)^#^	39 (15.0)	49 (16.6)	-1.6 (-4.7–7.9)
Two	148 (25.6)	45 (33.1)	90 (22.9)	10.2 (1.3–19.7)^#^	63 (24.0)	81 (27.4)	-3.4 (-4.2–10.7)
Three or more	338 (58.5)	39 (28.7)	269 (68.4)	-39.7 (30.0–48.3)^#^	160 (61.0)	166 (56.0)	5.0 (-3.5–13.0)
**Previous child death**^Ɨ^							
None	382 (78.6)	64 (76.2)	281 (78.3)	-2.1 (-7.5–13.6)	170 (76.2)	200 (81.0)	-4.8 (-3.0–12.5)
At least one	104 (21.4)	20 (23.8)	78 (21.7)	2.1 (-7.5–13.6)	53 (23.8)	47 (19.0)	4.8 (-3.0–12.5)
**Education level**							
None	55 (9.6)	15 (11.0)	39 (10.0)	1.0 (-4.5–8.4)	22 (8.4)	33 (11.2)	-2.8 (-2.5–8.1)
Primary	314 (54.5)	60 (44.1)	224 (57.3)	-13.2 (3.1–22.9)^#^	155 (59.2)	147 (50.0)	9.2 (0.6–17.5)^#^
At least secondary	207 (35.9)	61 (44.9)	128 (32.7)	12.2 (2.3–22.0)^#^	85 (32.4)	114 (38.8)	-6.4 (-1.9–14.4)
**Religion**							
Christianity	496 (87.0)	108 (80.0)	346 (89.4)	-9.4 (2.2–17.8)^#^	230 (88.1)	251 (86.9)	1.2 (-4.6–7.1)
Islam	44 (7.7)	15 (11.1)	28 (7.2)	3.9 (-1.8–11.1)	19 (7.3)	24 (8.3)	-1.0 (-3.9–5.8)
Other religion	30 (5.3)	12 (8.9)	13 (3.4)	5.5 (0.8–12.2)^#^	12 (4.6)	14 (4.8)	-0.2 (-3.8–4.2)
**HIV test in index pregnancy**							
No, already known HIV positive	42 (7.4)	6 (4.5)	31 (7.9)	-3.4 (-2.5–7.7)	17 (6.5)	23 (7.9)	-1.4 (-3.4–6.0)
Yes, tested for HIV	529 (92.6)	128 (95.5)	359 (92.1)	3.4 (-2.5–7.7)	243 (93.5)	268 (92.1)	1.4 (-3.4–6.0)
**HIV result if tested in index pregnancy**							
Negative	58 (11.0)	16 (12.5)	36 (10.0)	2.5 (-3.7–10.3)	33 (13.6)	21 (7.8)	5.8 (0.1–11.6)^#^
Positive	471 (89.0)	112 (87.5)	323 (90.0)	-2.5 (-3.7–10.3)	210 (86.4)	247 (92.2)	-5.8 (0.1–11.6)^#^
Disclosure of HIV status to partner^*^							
Yes	429 (81.6)	106 (82.8)	288 (80.9)	1.9 (-6.9–9.4)	195 (81.3)	220 (82.1)	-0.8 (-6.1–7.9)
No	67 (12.7)	16 (12.5)	44 (12.4)	0.1 (-6.2–8.1)	37 (15.4)	26 (9.7)	5.7 (-0.3–11.9)
No partner	30 (5.7)	6 (4.7)	24 (6.7)	-2.0 (-4.1–6.3)	8 (3.3)	22 (8.2)	-4.9 (0.4–9.4)^#^
**Known HIV status of partner**^§1^							
HIV status unknown	187 (34.1)	48 (36.6)	122 (33.2)	3.4 (-6.1–13.6)	86 (33.9)	95 (34.7)	-0.8 (-7.5–9.1)
HIV status known	361 (65.9)	83 (63.4)	246 (66.8)	-3.4 (-6.1–13.6)	168 (66.1)	179 (65.3)	0.8 (-7.5–9.1)
**Partner’s HIV result if known**^§2^							
HIV negative	99 (27.4)	29 (34.9)	62 (25.2)	9.7 (-1.9–22.2)	47 (28.0)	48 (26.8)	1.2 (-8.6–10.9)
HIV positive	262 (72.6)	54 (65.1)	184 (74.8)	-9.7 (-1.9–22.2)	121 (72.0)	131 (73.2)	-1.2 (-8.6–10.9)
**ART in previous pregnancy**^Ŧ1^							
No ART in pregnancy	255 (53.4)	48 (57.8)	183 (51.9)	5.9 (-6.5–17.9)	99 (45.0)	149 (61.6)	-16.6 (7.2–25.6)^#^
sdNVP in pregnancy	57 (11.9)	12 (14.5)	40 (11.3)	3.2 (-4.4–13.4)	28 (12.7)	28 (11.6)	1.1 (-5.1–7.6)
ART in pregnancy	166 (34.7)	23 (27.7)	130 (36.8)	-9.1 (-3.0–19.6)	93 (42.3)	65 (26.8)	15.5 (6.4–24.1)^#^
**Timing of ART**^Ŧ2^							
Start before pregnancy of index baby	250 (43.9)	43 (32.1)	191 (49.4)	-17.3 (7.2–26.5)^#^	128 (49.8)	118 (40.3)	9.5 (1.0–18.0)^#^
Start during pregnancy of index baby	258 (45.4)	75 (56.0)	158 (40.8)	15.2 (5.0–24.9)^#^	96 (37.4)	151 (51.5)	-14.1 (5.6–22.5)^#^
Never on ART	61 (10.7)	16 (11.9)	38 (9.8)	2.1 (-3.8–9.6)	33 (12.8)	24 (8.2)	4.6 (-0.7–10.2)
**Health status at ART initiation**							
No illness	407 (80.3)	93 (78.8)	283 (81.3)	-2.5 (-5.7–12.0)	170 (75.9)	224 (83.6)	-7.7 (0.3–15.2)^#^
A little bit sick	79 (15.6)	21 (17.8)	50 (14.4)	3.4 (-4.1–12.5)	44 (19.6)	33 (12.3)	7.3 (0.6–14.3)^#^
Very sick	21 (4.1)	4 (3.4)	15 (4.3)	-0.9 (-4.9–4.6)	10 (4.5)	11 (4.1)	0.4 (-3.6–4.7)
**Health status at enrolment**							
No illness	551 (95.7)	132 (97.1)	370 (94.6)	2.5 (-2.8–5.9)	247 (94.6)	284 (96.3)	-1.7 (-2.2–5.7)
A little bit sick	25 (4.3)	4 (2.9)	21 (5.4)	-2.5 (-2.8–5.9)	14 (5.4)	11 (3.7)	1.7 (-2.2–5.7)
Very sick	0 (0.0)	0 (0.0)	0 (0.0)		0 (0.0)	0 (0.0)	
**Future pregnancy intention**							
Yes	136 (25.7)				54 (21.4)	82 (29.6)	-8.2 (0.4–15.7)^#^
No	393 (74.3)				198 (78.6)	195 (70.4)	8.2 (0.4–15.7)^#^
**Current contraceptive use**							
Yes	262 (47.0)	54 (39.7)	198 (50.4)	-10.7 (0.5–20.3)^#^			
No	296 (53.0)	82 (60.3)	195 (49.6)	10.7 (0.5–20.3)^#^			

* Disclosure of HIV status to partner for only those with a known HIV result and with a partner. Chi-square *P* value excludes those in “No partner” category.

Ɨ Previous child deaths for those with at least two children.

Ŧ^1^ ART in previous pregnancies other than pregnancy of index baby. ^2^Timing of ART if they have been on ART ever since.

§^1^ Known HIV status of partner and ^2^partner HIV result if known ever since they became partners.

¶ Difference between proportions: future pregnancy intention (yes vs. no) or current contraceptive use (yes vs. no), and 95% Confidence Intervals (CI) with continuity correction.

# The difference between two proportions is statistically significant.

** Missing outcome data: future pregnancy intention (49), and current contraceptive use (20). Missing categorical variable data were excluded in analysis.

ART–Antiretroviral therapy, HIV–Human Immunodeficiency Virus, sdNVP–single dose Neverapine.

NOTE: all percentages were rounded off such that they sum to 100% in each column for each factor

### Factors associated with unintended index pregnancy and unmet contraceptive need

In multivariable analysis, unintended index pregnancy was associated with age category and parity. In women who were ≥35 years old, the odds of unintended pregnancy were twice as high as in women aged 14–24 years (adjusted OR [aOR]: 2.06, 95%CI: 1.01–4.19) and women with a parity of three or more deliveries had an almost three-fold increased odds of unintended index pregnancy compared to primiparous women (aOR: 2.89, 95%CI: 1.49–5.59) (Table 3, model 1).

**Table 3:** Factors associated with unintended index pregnancy and unmet contraceptive need for the index pregnancy as reported at 4–26 weeks post-partum.

	Model 1: Unintended index pregnancy				Model 2: Unmet contraceptive need for the index pregnancy**			
	OR	95% CI	aOR^ƗƗ^	95%CI	OR	95% CI	aOR^ŦŦ^	95%CI
**Age group (years)**								
14–24	1		1		4.24	(1.80–9.98) ^¶^	4.17	(1.76–9.87) ^¶^
25–34	1.43	(0.97–2.12)	0.70	(0.42–1.18)	1.45	(0.71–2.96)	1.41	(0.66–3.02)
≥ 35	5.45	(2.94–10.11) ^¶^	2.06	(1.01–4.19) ^¶^	1		1	
**Parity**								
One	1		1		16.53	(4.20–65.01) ^¶^	8.31	(1.75–39.47) ^¶^
Two	1.25	(0.69–2.26)	1.25	(0.67–2.32)	1.20	(0.58–2.46)	1.09	(0.46–2.60)
Three or more	3.22	(1.89–5.48) ^¶^	2.89	(1.49–5.59) ^¶^	1		1	
**Previous child death**^Ɨ^								
None	1				1			
At least one	1.08	(0.70–1.68)			1.11	(0.55–2.23)		
**Education level**								
None	0.84	(0.46–1.51)			0.85	(0.32–2.26)		
Primary	1				1			
At least secondary	0.89	(0.62–1.27)			0.78	(0.43–1.39)		
**Religion**								
Christianity	1				1			
Islam	0.86	(0.46–1.62)			1.75	(0.64–4.76)		
Other religion	0.91	(0.42–1.93)			0.66	(0.17–2.52)		
**HIV test in index pregnancy**								
No, already known HIV positive	0.69	(0.36–1.32)			1.66	(0.58–4.77)		
Yes, tested for HIV	1				1			
**HIV result if tested in index pregnancy**								
Negative	1				1			
Positive	1.62	(0.90–2.91)			0.86	(0.32–2.32)		
Disclosure of HIV status to partner^*^								
Yes	1				1			
No	1.05	(0.62–1.77)			1.58	(0.70–3.58)		
**Known HIV status of partner**^§1^								
HIV status unknown	1.02	(0.71–1.46)			2.34	(1.30–4.20) ^¶^	2.17	(1.19–3.95) ^¶^
HIV status known	1				1		1	
**Partner’s HIV result if known**^§2^								
HIV negative	1		1		1			
HIV positive	1.71	(1.05–2.80) ^¶^	1.56	(0.92–2.65)	1.40	(0.55–3.57)		
**ART in previous pregnancy**^Ŧ1^								
No ART in pregnancy	1				1		1	
sdNVP in pregnancy	1.13	(0.63–2.01)			0.65	(0.25–1.70)	0.68	(0.25–1.87)
ART in pregnancy	1.22	(0.82–1.81)			0.49	(0.25–0.97) ^¶^	0.62	(0.31–1.26)
**Timing of ART**^Ŧ2^								
Start before pregnancy of index baby	1.90	(1.05–3.44) ^¶^	1.59	(0.85–2.98)	1			
Start during pregnancy of index baby	0.99	(0.55–1.79)	0.96	(0.52–1.79)	1.59	(0.90–2.81)		
Never on ART	1		1		1.37	(0.52–3.60)		
**Health status at ART initiation**								
No illness	1				1			
A little bit sick	1.53	(0.94–2.49)			0.86	(0.41–1.81)		
Very sick	1.99	(0.82–4.85)			0.60	(0.15–2.31)		
**Health status at enrolment**								
No illness	1				1			
A little bit sick	0.77	(0.33–1.77)			0.93	(0.22–3.81)		

* Disclosure of HIV status to partner for only those with a known HIV result and with a partner.

Ɨ Previous child deaths for those with at least two children.

Ŧ^1^ART in previous pregnancies other than pregnancy of index baby. ^2^Timing of ART if they have been on ART ever since.

§^1^Known HIV status of partner and ^2^partner HIV result if known ever since they became partners.

¶ Significant odds ratio 95% confidence intervals.

** Women who want to stop or delay childbearing but are not using any method of contraceptives (World Health Organization definition).

ƗƗ Variables in model 1 are adjusted for age, parity, partner’s HIV result if known and timing of ART.

ŦŦ Variables in model 2 are adjusted for age, known HIV status of partner and ART in previous pregnancy.

aOR–adjusted Odds Ratio, ART- Antiretroviral Therapy, CI–Confidence Interval, HIV–Human Immunodeficiency Virus, sdNVP–single dose Neverapine.

Unmet contraceptive need for the index pregnancy was independently associated with age category, parity and ignorance of the HIV status of the partner. The odds of unmet contraceptive need for the index pregnancy were 4 times higher in women aged 14–24 than in women who were ≥35 years old (aOR: 4.17, 95%CI: 1.76–9.87), 8 times higher in primiparous than in women with a parity of three or more (aOR: 8.31, 95%CI: 1.75–39.47) and twice as high in women whose partner had an unknown HIV status as in those with a partner with a known HIV status (aOR: 2.17, 95%CI: 1.19–3.95) (Table 3, model 2).

### Factors associated with future pregnancy intention and current contraceptive use

Future pregnancy intention was associated with age category, parity and religious affiliation in multivariable analysis. In women aged 14–24 years, the odds to desire a future pregnancy were 3 times higher than in women who were ≥35 years old (aOR: 3.20, 95%CI: 1.10–9.31). While the odds for primiparous women to desire a future pregnancy were seven times higher than women with parity of three or more children (aOR: 7.34, 95%CI: 3.74–14.40), secundiparous women had twice the odds of future pregnancy intention compared to women with parity of three or more children (aOR: 2.65, 95%CI: 1.53–4.59). Women of religion other than Christianity or Islam had double odds to desire a future pregnancy (aOR: 2.75, 95%CI: 1.11–6.80) (Table 4, model 3).

**Table 4:** Factors associated with future pregnancy intentions and current contraceptive use as reported at 4–26 weeks post-partum.

	Model 3: Future pregnancy intention				Model 4: Current contraceptive use			
	OR	95% CI	aOR^ƗƗ^	95%CI	OR	95% CI	aOR^ŦŦ^	95%CI
**Age group (years)**								
14–24	11.19	(3.97–31.56) ^¶^	3.20	(1.10–9.31) ^¶^	1			
25–34	3.45	(1.32–9.04) ^¶^	2.44	(0.92–6.45)	1.23	(0.84–1.80)		
≥ 35	1		1		1.22	(0.72–2.08)		
**Parity**								
One	10.55	(5.67–19.62) ^¶^	7.34	(3.74–14.40) ^¶^	0.83	(0.51–1.33)		
Two	3.45	(2.08–5.72) ^¶^	2.65	(1.53–4.59) ^¶^	0.81	(0.54–1.20)		
Three or more	1		1		1			
**Previous child death**^Ɨ^								
None	1				1			
At least one	1.13	(0.64–1.97)			1.33	(0.85–2.07)		
**Education level**								
None	1.44	(0.74–2.78)	1.69	(0.81–3.52)	0.63	(0.35–1.14)		
Primary	1		1		1			
At least secondary	1.78	(1.17–2.71) ^¶^	1.44	(0.91–2.30)	0.71	(0.49–1.02)		
**Religion**								
Christianity	1		1		1			
Islam	1.72	(0.88–3.34)	1.43	(0.68–3.01)	0.86	(0.46–1.62)		
Other religion	2.96	(1.30–6.72) ^¶^	2.75	(1.11–6.80) ^¶^	0.94	(0.42–2.07)		
**HIV test in index pregnancy**								
No, already known HIV positive	1.84	(0.75–4.53)			1.23	(0.64–2.35)		
Yes, tested for HIV	1				1			
**HIV result if tested in index pregnancy**								
Negative	1				1		1	
Positive	0.78	(0.42–1.46)			0.54	(0.30–0.97) ^¶^	1.76	(0.28–11.1)
Disclosure of HIV status to partner^*^								
Yes	1				1			
No	0.99	(0.53–1.83)			1.61	(0.94–2.75)		
**Known HIV status of partner**^§1^								
HIV status unknown	1.17	(0.77–1.77)			0.96	(0.67–1.38)		
HIV status known	1				1			
**Partner’s HIV result if known**^§2^								
HIV negative	1				1			
HIV positive	0.63	(0.37–1.07)			0.94	(0.59–1.51)		
**ART in previous pregnancy**^Ŧ1^								
No ART in pregnancy	1				1		1	
sdNVP in pregnancy	1.14	(0.56–2.35)			1.51	(0.84–2.70)	1.85	(0.94–3.63)
ART in pregnancy	0.67	(0.39–1.17)			2.15	(1.42–3.26) ^¶^	2.45	(1.52–3.93) ^¶^
**Timing of ART**^Ŧ2^								
Start before pregnancy of index baby	1		1		1		1	
Start during pregnancy of index baby	2.11	(1.36–3.26) ^¶^	1.56	(0.97–2.49)	0.59	(0.41–0.84) ^¶^	0.84	(0.53–1.36)
Never on ART	1.87	(0.95–3.68)	1.11	(0.52–2.34)	1.27	(0.71–2.27)	-	-
**Health status at ART initiation**								
No illness	1				1		1	
A little bit sick	1.28	(0.73–2.24)			1.76	(1.07–2.89) ^¶^	1.79	(0.98–3.26)
Very sick	0.81	(0.26–2.51)			1.20	(0.50–2.89)	3.01	(0.85–10.58)
**Health status at enrolment**								
No illness	1				1			
A little bit sick	0.53	(0.18–1.59)			1.46	(0.65–3.29)		
**Future pregnancy intention**								
Yes					1		1	
No					1.54	(1.03–2.30) ^¶^	1.81	(1.03–3.16) ^¶^

* Disclosure of HIV status to partner for only those with a known HIV result and with a partner.

Ɨ Previous child deaths for those with at least two children.

Ŧ^1^ART in previous pregnancies other than pregnancy of index baby. ^2^Timing of ART if they have been on ART ever since.

§^1^Known HIV status of partner and ^2^partner HIV result if known ever since they became partners.

¶ Significant odds ratio 95% confidence intervals.

ƗƗ Variables in model 3 are adjusted for age, parity and education level, religion and timing of ART.

ŦŦ Variables in model 4 are adjusted for HIV result if tested in index pregnancy, ART in previous pregnancy, Timing of ART, health status at ART initiation, future pregnancy intention.

- Omitted because of collinearity with other terms in the model.

aOR–adjusted Odds Ratio, ART- Antiretroviral therapy, CI–confidence interval, HIV–Human Immunodeficiency Virus, sdNVP–single dose Neverapine.

Current contraceptive use was independently associated with history of ART and a woman’s future pregnancy intention. The odds of current contraceptive use among women who were on a triple drug ART regimen in the previous pregnancy were two times more than those who had never been on ART in previous pregnancy (aOR: 2.45, 95%CI: 1.52–3.93), whereas women without future pregnancy intentions had nearly double odds of being on a contraceptive as those with future pregnancy intentions (aOR: 1.81, 95%CI: 1.03–3.16) (Table 4, model 4).

## Discussion

Among Malawian women living with HIV who were enrolled in this study between 4 to 26 weeks after delivery, we observed that 2 in 5 self-reported unintended index pregnancy, 3 in 10 reported unmet contraceptive need at the time of index pregnancy, 1 in 4 reported future pregnancy intention, and 1 in 2 reported current contraceptive use among those without pregnancy intentions within the next 12 months. Older age and higher parity were associated with unintended pregnancy, whereas younger age and lower parity were associated with both unmet contraceptive need and future pregnancy intention. Unmet contraceptive need was also associated with unknown HIV status of partner, and current contraceptive use with being on ART in the index pregnancy.

The high prevalence of unintended index pregnancy and unmet contraceptive need reported in this study are comparable to studies in sub-Saharan Africa, with prevalence of unintended pregnancy ranging between 35.1%-69.0% and unmet contraceptive need between 36.0%-52.2% [19–21,23]. Our results underline the need to improve Malawian women’s access to effective and appropriate contraceptive methods in order to minimize unintended pregnancies in the general population, particularly in women living with HIV where this is an important strategy to achieving virtual elimination of MTCT [7].

The factors that we found to be associated with unintended index pregnancy (higher age and higher parity) and with future pregnancy intention (younger age and lower parity) suggest areas for targeted interventions to reduce unintended pregnancies among women living with HIV. Primiparous and young women are likely to want more pregnancies, especially in a low-income country like Malawi, due to spousal, familial, societal and/or cultural pressures [26]. Additionally, women living with HIV with pregnancy intention in the era of Option B+ may be confident in the knowledge that ART will maintain their good health status and prevent vertical transmission [26,27]. Our data suggest that targeted contraceptive counselling for all women living with HIV of higher age and parity may be particularly effective in preventing unintended pregnancies. In addition, preconception counselling for younger women with future pregnancy intentions may improve timing of future pregnancies aligning with viral load levels to ensure lowest risk of vertical transmission.

We did not find associations between future pregnancy intentions and a women’s HIV test result during index pregnancy, disclosure of her HIV status to her partner or knowledge of the partner’s HIV status. This is contrary to earlier studies from the pre-‘Option B+’ era, which demonstrated that Malawian women had lower pregnancy intentions if they knew they were HIV positive [8,27]. Our findings suggest that since the introduction of Option B+, a couple’s HIV status may no longer strongly determine women’s pregnancy intentions, likely due to knowledge about how ART would prevent vertical transmission, which is supported by results from a recent qualitative study from Malawi [26].

The associations between unmet contraceptive need and younger age and primiparity in our study population add to a growing body of literature reporting challenges that adolescent girls and young women face in accessing family planning and engaging with PMTCT programs in sub-Saharan Africa. Issues such as late diagnosis of HIV, practical demands of taking medication, cultural norms, travel distance to the clinic, limited social support, negative interactions with health care workers and stigma have all been found to contribute to limited uptake of PMTCT and family planning programs among adolescent girls and young women [28–30].These challenges may be mitigated by interventions such as short phone message reminders for medication, changing health care worker attitudes, psychological counselling, campaigns to improve HIV knowledge and early diagnosis, and financial and social support to families [30]. Such interventions may improve access to comprehensive antenatal, PMTCT and family planning services, thereby preventing unwanted pregnancies as well as MTCT. Integration of family planning services into HIV care services has also shown to increase access to a wider range of family planning methods [12,14,31]. However, the potential negative unintended consequences of integration on health care systems must be acknowledged and addressed [14,15].

Only a few women whose index pregnancy was unintended and who reported their partner’s HIV status as negative, reportedly used condoms. Previous research in Malawi has attributed such scenarios to partners’ refusal to use condoms [19]. This highlights the importance of contraceptive counselling among discordant couples, with emphasis on prevention of horizontal transmission of HIV. Additionally, unmet contraceptive need was higher among those with unknown HIV status of a male partner, likely due to non-disclosure of HIV status or the partner not testing for HIV. Non-disclosure of a male partner’s HIV status has been linked to fears of divorce, being blamed for infidelity, stigma, poverty, disharmony and denial [32,33]. Barriers to HIV testing among male partners have been described qualitatively, and include stigma, discrimination, time constraints, lack of confidentiality, sociocultural norms and fears of blame and divorce [34]. Targeted family counselling by trusted health workers is likely to promote disclosure of HIV status among couples [32], partner referral in PMTCT [35], and index case testing. Community-based HIV testing programs, such as HIV self-testing, will not only encourage disclosure but also increase male partners’ involvement in HIV testing [34,36,37].

We observed that slightly less than a quarter of women in this cohort desired a future pregnancy, which may relate to the timing of our enrolment in the period soon after delivery. Among those with future pregnancy intentions, almost all wanted to delay their next pregnancy for at least12 months, but contraceptive use among these women poorly matched their future pregnancy intentions. Our prevalence of unmet postpartum contraceptive need (49.6%) is consistent with reports from Guatemala (67.6%), Western Kenya (46.0%), and India (64.0%) [22]. Family planning services specific to postpartum women should be considered in high fertility settings like Malawi. Our further observation that women who were on ART in the previous pregnancy had increased current contraceptive uptake may relate to their exposure to contraceptive messages during multiple ART visits. Integration of HIV family planning services in HIV care, starting directly after diagnosis, must be fully supported to meet family planning goals for all women living with HIV.

Nearly two-thirds of the women with unintended index pregnancy reported using contraceptives at time of conception. Future studies should explore reasons for the high reported contraceptive failure among women in Option B+. Multiple reasons may explain this finding, such as interruption of contraceptive service provision, discontinuation of contraceptives, sub-optimal adherence and poor knowledge about contraceptive usage [20]. In addition, over half of the women with unintended pregnancy while on contraceptives were on ART. Contraceptive failure may be caused by drug interactions between efavirenz and nevirapine-containing ART regimens and combination hormonal oral contraceptives, and between efavirenz and implant-levonorgestrel, although the impact of the latter interaction is subject to debate [38–40].

The strengths of our study include the large sample size of HIV-positive women and the comprehensive description of pregnancy intentions and contraceptive use in Malawian women, the first conducted in the Option B+ era. However, a number of limitations also need to be highlighted. We modestly measured unintended pregnancy, whereas a detailed measure would have been more ideal if it were not for increased interview time which would have rendered participant loss. We also enrolled women from 3 large patient volume government health facilities; therefore, our results may not be representative for populations in private/religious-based or smaller health facilities. In addition, our data were pooled across the three sites representing urban and rural settings, and site population effects were not studied. However, similar proportions of contraceptive use (63.1% v 58.5%) and unmet contraceptive need (16.1% v 19.2%) for urban and rural settings respectively have been reported in the 2015–2016 Malawi Demographic Health Survey reports [4]. Since our study population was limited to women presenting at under-5 clinics, women whose child died or who did not visit an under-5 clinic were not included; however, the under-5 coverage in Malawi is high, at 95% [4]. In addition, the cross-sectional nature of the study restricts drawing causal inferences about the associations observed between individual characteristics and outcomes. We report about data that were collected 4–26 weeks after delivery, while women’s views on pregnancy intention and contraceptives can be complex and dynamic [27,41], and may differ in relation to the time since the last delivery. Furthermore, retrospective questioning about pregnancy intention and contraceptive use before the index pregnancy is sensitive to recall bias. Our data collection tool did not discriminate between unwanted and mistimed unintended index pregnancy, and there may be different reasons and associated factors for each. Similarly, the duration of time since delivery could have an impact on the need for contraception, as women may have less sexual activity shortly after giving birth, however the proportion of women who were enrolled 12–26 weeks post-delivery was small and we therefore did not include infant age as a variable in the model for current contraceptive use. Finally, the large majority of women had a partner, but we only observed pregnancy intentions and contraceptive use reported by women. Expectations of spouses, families and society that are known to impact family planning decisions were not studied [26,27].

## Conclusions

High prevalence of unintended index pregnancy and of unmet need for contraception were observed among Malawian women living with HIV in the Option B+ era. This highlights the need for improved access to contraceptive and family planning services. To help achieve reproductive goals and elimination of MTCT of HIV in Malawi, integration of family planning services into HIV care should be strengthened to ensure all women living with HIV have timely access to a wide range of family planning methods which have low failure risk.

## Supporting information

S1 Appendix Structured questionnaire for pregnancy intention and contraceptive use. (PDF)

## References

[1] UNAIDS. Global HIV & AIDS statistics—2018 fact sheet [Internet]. 2018 [cited 7 Mar 2019]. Available: http://www.unaids.org/en/resources/fact-sheet

[2] National AIDS Commission. The Malawi National HIV and AIDS Strategic Plan 2011–2016 [Internet]. NAC; 2012. Available: http://www.emtct-iatt.org/wp-content/uploads/2016/01/Malawi-HIV-Strategic-Plan-2011-2016.pdf

[3] UNGASS. Malawi HIV and AIDS Monitoring and Evaluation Report: 2008–2009 [Internet]. UNGASS; 2010 Mar. Available: http://data.unaids.org/pub/Report/2010/malawi_2010_country_progress_report_en.pdf

[4] National Statistical Office, Zomba, Malawi and ICF Macro, Calverton, Maryland, USA. The Malawi Demographic Health Survey 2015–16 [Internet]. Malawi; 2016 May. Available: http://www.nsomalawi.mw/images/stories/data_on_line/demography/mdhs2015_16/MDHS%202015-16%20Final%20Report.pdf

[5] Center for Disease Control and Prevention. Impact of an Innovative Approach to Prevent Mother-to-Child Transmission of HIV in Malawi [Internet]. Malawi; 2011 Jul. Available: https://www.cdc.gov/mmwr/preview/mmwrhtml/mm6208a3.htm

[6] Barr BTA, Schouten E, Oosterhout J van, Gupta SK, Phiri H, Thindwa D, et al. National HIV Transmission in 4–12 Week Olds in Malawi’s PMTCT Option B+ Program | CROI Conference [Internet]. [cited 4 Aug 2017]. Available: http://www.croiconference.org/sessions/national-hiv-transmission-4-12-week-olds-malawi%E2%80%99s-pmtct-option-b-program

[7] WHO | Elimination of mother-to-child transmission (EMTCT) of HIV and syphilis. In: WHO [Internet]. [cited 24 Apr 2017]. Available: http://www.who.int/reproductivehealth/publications/rtis/9789241505888/en/

[8] Hoffman IF, Martinson FEA, Powers KA, Chilongozi DA, Msiska ED, Kachipapa EI, et al The Year-Long Effect of HIV-Positive Test Results on Pregnancy Intentions, Contraceptive Use, and Pregnancy Incidence Among Malawian Women: JAIDS J Acquir Immune Defic Syndr. 2008;47: 477–483. 10.1097/QAI.0b013e318165dc52 18209677

[9] WHO. Strategic Approaches to the Prevention of HIV Infection in Infants [Internet]. Morges, Switzerland; 2002 Mar p. Section 4.2.10. Available: http://www.who.int/hiv/pub/mtct/en/StrategicApproachesE.pdf

[10] Hladik W, Stover J, Esiru G, Harper M, Tappero J. The Contribution of Family Planning towards the Prevention of Vertical HIV Transmission in Uganda. PLOS ONE. 2009;4: e7691 10.1371/journal.pone.0007691 19888347 PMC2766039

[11] Tsui AO, McDonald-Mosley R, Burke AE. Family Planning and the Burden of Unintended Pregnancies. Epidemiol Rev. 2010;32: 152–174. 10.1093/epirev/mxq012 20570955 PMC3115338

[12] Cohen CR, Grossman D, Onono M, Blat C, Newmann SJ, Burger RL, et al Integration of family planning services into HIV care clinics: Results one year after a cluster randomized controlled trial in Kenya. PLOS ONE. 2017;12: e0172992 10.1371/journal.pone.0172992 28328966 PMC5362197

[13] Haberlen SA, Narasimhan M, Beres LK, Kennedy CE. Integration of Family Planning Services into HIV Care and Treatment Services: A Systematic Review. Stud Fam Plann. 2017;48: 153–177. 10.1111/sifp.12018 28337766 PMC5516228

[14] Phiri S, Feldacker C, Chaweza T, Mlundira L, Tweya H, Speight C, et al Integrating reproductive health services into HIV care: strategies for successful implementation in a low-resource HIV clinic in Lilongwe, Malawi. J Fam Plann Reprod Health Care. 2015; jfprhc-2013-100816. 10.1136/jfprhc-2013-100816 25902815 PMC4717379

[15] Siapka M, Obure CD, Mayhew SH, Sweeney S, Fenty J, Initiative I, et al Impact of integration of sexual and reproductive health services on consultation duration times: results from the Integra Initiative. Health Policy Plan. 2017;32: iv82–iv90. 10.1093/heapol/czx141 29194545 PMC5886289

[16] Tweya H, Feldacker C, Gugsa S, Phiri S. Contraceptive use and pregnancy rates among women receiving antiretroviral therapy in Malawi: a retrospective cohort study. Reprod Health. 2018;15: 25 10.1186/s12978-017-0440-0 29426333 PMC5807743

[17] Department of HIV/AIDS. Malawi Guidelines for Clinical Management of HIV in Children and Adults | 2016 [Internet]. 2015 [cited 13 Dec 2018]. Available: https://aidsfree.usaid.gov/resources/malawi-guidelines-clinical-management-hiv-children-and-adults

[18] Tweya H, Feldacker C, Haddad LB, Munthali C, Bwanali M, Speight C, et al Integrating family planning services into HIV care: use of a point-of-care electronic medical record system in Lilongwe, Malawi. Glob Health Action. 2017;10: 1383724 10.1080/16549716.2017.1383724 29039263 PMC5678434

[19] Haddad LB, Feldacker C, Jamieson DJ, Tweya H, Cwiak C, Chaweza T, et al Pregnancy Prevention and Condom Use Practices among HIV-Infected Women on Antiretroviral Therapy Seeking Family Planning in Lilongwe, Malawi. PLOS ONE. 2015;10: e0121039 10.1371/journal.pone.0121039 25811849 PMC4374940

[20] Mayondi GK, Wirth K, Morroni C, Moyo S, Ajibola G, Diseko M, et al Unintended pregnancy, contraceptive use, and childbearing desires among HIV-infected and HIV-uninfected women in Botswana: across-sectional study. BMC Public Health. 2016;16: 44 10.1186/s12889-015-2498-3 26774918 PMC4715872

[21] McCoy SI, Buzdugan R, Ralph LJ, Mushavi A, Mahomva A, Hakobyan A, et al Unmet Need for Family Planning, Contraceptive Failure, and Unintended Pregnancy among HIV-Infected and HIV-Uninfected Women in Zimbabwe. PLOS ONE. 2014;9: e105320 10.1371/journal.pone.0105320 25144229 PMC4140753

[22] Pasha O, Goudar SS, Patel A, Garces A, Esamai F, Chomba E, et al Postpartum contraceptive use and unmet need for family planning in five low-income countries. Reprod Health. 2015;12: S11 10.1186/1742-4755-12-S2-S11 26063346 PMC4464604

[23] O’Shea MS, Rosenberg NE, Hosseinipour MC, Stuart GS, Miller WC, Kaliti SM, et al Effect of HIV status on fertility desire and knowledge of long-acting reversible contraception of postpartum Malawian women. AIDS Care. 2015;27: 489–498. 10.1080/09540121.2014.972323 25367269 PMC4312510

[24] Mandiwa C, Namondwe B, Makwinja A, Zamawe C. Factors associated with contraceptive use among young women in Malawi: analysis of the 2015–16 Malawi demographic and health survey data. Contracept Reprod Med. 2018;3: 12 10.1186/s40834-018-0065-x 30250748 PMC6146597

[25] Johnsson T. A procedure for stepwise regression analysis. Stat Pap. 1992;33: 21–29. 10.1007/BF02925308

[26] Biseck T, Kumwenda S, Kalulu K, Chidziwisano K, Kalumbi L. Exploring fertility decisions among pregnant HIV-positive women on antiretroviral therapy at a health centre in Balaka, Malawi: A descriptive qualitative. Malawi Med J. 2015;27: 128–134. 26955433 PMC4761703

[27] Taulo F, Berry M, Tsui A, Makanani B, Kafulafula G, Li Q, et al Fertility Intentions of HIV-1 Infected and Uninfected Women in Malawi: A Longitudinal Study. AIDS Behav. 2009;13: 20–27. 10.1007/s10461-009-9547-9 19308718

[28] Horwood C, Butler LM, Haskins L, Phakathi S, Rollins N. HIV-Infected Adolescent Mothers and Their Infants: Low Coverage of HIV Services and High Risk of HIV Transmission in KwaZulu-Natal, South Africa. PLOS ONE. 2013;8: e74568 10.1371/journal.pone.0074568 24073215 PMC3779214

[29] Landes M, Sodhi S, Matengeni A, Meaney C, van Lettow M, Chan AK, et al Characteristics and outcomes of women initiating ART during pregnancy versus breastfeeding in Option B+ in Malawi. BMC Public Health. 2016;16: 713 10.1186/s12889-016-3380-7 PMC4973045 27487775

[30] Ronen K, McGrath CJ, Langat ACMbc, Kinuthia JMbc, Omolo D, Singa BMbc, et al Gaps in Adolescent Engagement in Antenatal Care and Prevention of Mother-to-Child HIV Transmission Services in Kenya. [Miscellaneous Article]. J Acquir Immune Defic Syndr. 2017;74: 30–37. 10.1097/QAI.0000000000001176 27599005 PMC5895459

[31] Tweya H, Feldacker C, Gugsa S, Phiri S. Contraceptive use and pregnancy rates among women receiving antiretroviral therapy in Malawi: a retrospective cohort study. Reprod Health. 2018;15 10.1186/s12978-017-0440-0 29426333 PMC5807743

[32] Walcott MM, Hatcher AM, Kwena Z, Turan JM. Facilitating HIV status disclosure for pregnant women and partners in rural Kenya: a qualitative study. BMC Public Health. 2013;13: 1115 10.1186/1471-2458-13-1115 24294994 PMC3907031

[33] Anglewicz P, Chintsanya J. Disclosure of HIV status between spouses in rural Malawi. AIDS Care. 2011;23: 998–1005. 10.1080/09540121.2010.542130 21390889 PMC3371657

[34] Choko AT, Kumwenda MK, Johnson CC, Sakala DW, Chikalipo MC, Fielding K, et al Acceptability of woman-delivered HIV self-testing to the male partner, and additional interventions: a qualitative study of antenatal care participants in Malawi. J Int AIDS Soc. 2017;20 10.7448/ias.20.1.21610 28691442 PMC5515040

[35] Elias M, Mmbaga EJ, Mohammed AA, Kishimba RS. Male partner involvement in the prevention of mother to child transmission of HIV infection in Mwanza Region, Tanzania. Pan Afr Med J. 2017;27 10.11604/pamj.2017.27.90.8901 28819511 PMC5554662

[36] Sharma M, Barnabas RV, Celum C. Community-based strategies to strengthen men’s engagement in the HIV care cascade in sub-Saharan Africa. PLOS Med. 2017;14: e1002262 10.1371/journal.pmed.1002262 28399122 PMC5388461

[37] Choko AT, MacPherson P, Webb EL, Willey BA, Feasy H, Sambakunsi R, et al Uptake, Accuracy, Safety, and Linkage into Care over Two Years of Promoting Annual Self-Testing for HIV in Blantyre, Malawi: A Community-Based Prospective Study. PLOS Med. 2015;12: e1001873 10.1371/journal.pmed.1001873 26348035 PMC4562710

[38] Pyra M, Heffron R, Mugo NR, Nanda K, Thomas KK, Celum C, et al Effectiveness of Hormonal Contraception in HIV-Infected Women using Antiretroviral Therapy: A Prospective Study. AIDS Lond Engl. 2015;29: 2353–2359. 10.1097/QAD.0000000000000827 26544706 PMC4748843

[39] Patel RC, Onono M, Gandhi M, Blat C, Hagey J, Shade SB, et al Pregnancy rates in HIV-positive women using contraceptives and efavirenz-based or nevirapine-based antiretroviral therapy in Kenya: a retrospective cohort study. Lancet HIV. 2015;2: e474–e482. 10.1016/S2352-3018(15)00184-8 26520927 PMC4632202

[40] Robinson JA, Jamshidi R, Burke AE. Contraception for the HIV-Positive Woman: A Review of Interactions between Hormonal Contraception and Antiretroviral Therapy. Infect Dis Obstet Gynecol. 2012;2012: e890160 10.1155/2012/890160 22927715 PMC3426212

[41] Huber S, Esber A, Garver S, Banda V, Norris A. The Relationship Between Ambivalent and Indifferent Pregnancy Desires and Contraceptive Use Among Malawian Women. Int Perspect Sex Reprod Health. 2017;43: 13–19. 10.1363/43e3417 28930624

