# Correction: Pregnancy intention and contraceptive use among HIV-positive Malawian women at 4-26 weeks post-partum: A nested cross-sectional study

**DOI:** 10.1371/journal.pone.0217330

**Published:** 2019-05-23

**Authors:** Deus Thindwa, Megan Landes, Monique van Lettow, Annie Kanyemba, Ernest Nkhoma, Happy Phiri, Thokozani Kalua, Joep J. van Oosterhout, Evelyn J. Kim, Beth A. Tippett Barr, on behalf of the NEMAPP Consortium

The third and eighth authors’ initials are indexed incorrectly in PubMed. The correct initials are: van Lettow M and van Oosterhout JJ. As a result, the citation in the published article is incorrect. The correct citation is: Thindwa D, Landes M, van Lettow M, Kanyemba A, Nkhoma E, Phiri H, et al. (2019) Pregnancy intention and contraceptive use among HIV-positive Malawian women at 4–26 weeks post-partum: A nested cross-sectional study. PLoS ONE 14(4): e0215947. https://doi.org/10.1371/journal.pone.0215947

## References

[1] Thindwa D, Landes M, Lettow Mv, Kanyemba A, Nkhoma E, Phiri H, et al (2019) Pregnancy intention and contraceptive use among HIV-positive Malawian women at 4–26 weeks post-partum: A nested cross-sectional study. PLoS ONE 14(4): e0215947 10.1371/journal.pone.0215947 31013338 PMC6478345

