# Supplementary material for: Pregnancy intention and contraceptive use among HIV-positive Malawian women at 4-26 weeks post-partum: A nested cross-sectional study
Source: PLoS One. 2019 Apr 23;14(4):e0215947. doi: 10.1371/journal.pone.0215947 (PMC6478345; doi:10.1371/journal.pone.0215947)
Supplement: S1 Appendix — (PDF) [file pone.0215947.s001.pdf]

NP01: NEMaPP SCREENING QUESTIONNAIRE

S

S01 Participant identification sticker — PLACE MOTHER STICKER HERE

S02 Date of screening: [ ][ ] d d / [ ][ ][ ] m m m / 2 0 1 [ ] y y y y

S03 Staff ID: [ ][ ][ ]

S04 Mother's ANC number *(leave blank if not found)*: [ ][ ][ ][ ][ ][ ]

S05 Parity *(=number of times mother has given birth)*: [ ][ ]

S06 Mother's birthdate: [ ][ ] d d / [ ][ ][ ] m m m / [ ][ ][ ][ ] y y y y

S07 Age of the mother: [ ][ ] *years*

S08 Infant/child's birthdate: [ ][ ] d d / [ ][ ][ ] m m m / [ ][ ][ ][ ] y y y y

S09 Sex of the child
Child1 ☐ Male ☐ Female
Child2 ☐ Male ☐ Female
Child3 ☐ Male ☐ Female

S10 Is the biological mother alive and here today?
Kodi ndinu kholo lomubereka mwanayu?
Ngati Ayi, kholo lake lili kuti?
- ☐ Mother is NOT alive
- ☐ Mother is ALIVE and here
- ☐ Mother is ALIVE but not here (STOP!!)

S11 Is the child less than 4 weeks of age?
Kodi mwanayu sanakwane masabata anayi?
[werengani kuchokera pa tsiku la kubadwa la mwana]
- ☐ No, the child is NOT younger than 4 weeks
- ☐ Yes, the child is younger than 4 weeks (STOP!!)

S12 Is the child aged 6 months or above?
Kodi mwanayu ali ndi miyezi isanu ndi umodzi kapena kupitilira?
[werengani kuchokera pa tsiku la kubadwa la mwana]
- ☐ No, the child is not older than 6 months
- ☐ Yes, the child is older than 6 months (STOP!!)

S13 Did you (the mother) go to ANC when you were pregnant?
Kodi panthawi yomwe munali oyembekezela mumapita kusikelo ya amayi oyembekezela?
- ☐ No
- ☐ Don't know
- ☐ Yes

S14 If YES; ANC site: [Ngati yankho ndi eya], Mumapita kuti:
[ ]

S15 Were you (the mother) tested during this last pregnancy?
Kodi munayezedwa magazi kuti mudziwe ngati muli ndikachilombo ka HIV pamene munali oyembekezela mimba yapitayi?
- ☐ No, already known HIV-positive
- ☐ Not tested for HIV, other reasons
- ☐ Yes, was tested in last pregnancy

S16 What was the result of last HIV test (the mother) received during this last pregnancy?
Kodi zotsatira za kuyeza HIV zimene munalandira komaliza pa mimba yapitayi zinali zotani?
☐ Negative ☐ Not willing to reveal / don't know
☐ Positive ☐ Not tested

S17 Are you (mother or caregiver) on ART now?
Kodi mukumwa ma ARV?
☐ No ☐ Not willing to reveal
☐ Yes

S18 If Yes, when did you first start ART?
[Ngati Eya],Kodi munayamba liti kumwa ma ARV?
☐ Not applicable
☐ Before this pregnancy
☐ During 1st or 2nd trimester (1-6 mos)
☐ 3rd trimester (7+ mos)
☐ Post-partum
☐ Don't know / not willing to reveal

STAFF SAYS; We will now take a blood sample to send to the lab to test the child for HIV. We will also take a sample from you (the mother/caregiver) to test your blood now and send to the lab for confirmation. Do you have questions before we start?

Tsopano titenga magazi amwana wanu kuti titumize ku lab kuti tikayeze HIV. Titenganso magazi anu(mayi/kapena oyang'anira mwana)kuti tiyese tsopano komanso titumize ku lab kuti titsimikize ngati muli ndi kachilombo ka HIV kapena ayi. Kodi muli ndi mafunso tisanayambe?

What is the mother's (or caregiver's) rapid HIV test result today? *(follow MoH guidelines)*

S19 [DETERMINE] ☐ Negative ☐ Positive ☐ Not tested

S20 [UNIGOLD] *(if Determine is reactive)* ☐ Negative ☐ Positive* ☐ Not tested

What is the mother's (or caregiver's) rapid HIV test result on same day parallel tests ? *(if first test inconclusive)*

S21 [DETERMINE] ☐ Negative ☐ Positive ☐ Not tested

S22 [UNIGOLD] ☐ Negative ☐ Positive* ☐ Not tested

If results from same day parallel tests were inconclusive, ask the participant to return after 4 weeks.
Put the screening form in the "inconclusive" box.

What is the mother's (or caregiver's) rapid HIV test result after 4 weeks? *(if parallel testing during enrolment was inconclusive)*

S23 [DETERMINE] ☐ Negative ☐ Positive ☐ Not tested

S24 [UNIGOLD] ☐ Negative ☐ Positive* ☐ Not tested

S25 If mother's rapid test result is positive now, but reported negative during ANC in last pregnancy (S16), check ANC register or Health Passport Book for ANC HIV test result.
☐ Not applicable. *(No need to answer S26,S27)*
☐ Not found
☐ Confirmed HTC result

S26 If confirmed HTC result in ANC register or Health Passport Book, result:
☐ Negative
☐ Positive

S27 If confirmed HTC result in ANC register or Health Passport Book, date of result:
☐☐ ☐☐☐ ☐☐☐☐
d d m m m y y y y

*** If rapid test (new) positive**
1. Provide post test counselling
2. Obtain consent to include mother and child in the 24 month follow up study
3. If consented to be included in the follow up study, administer enrolment form

Tell ALL mothers/caregivers that they will receive the results of the laboratory tests during the next EPI visit.
If that is not possible (i.e, if the next EPI visit is several months away, project staff will need to find a suitable time for a follow up visit. Mothers/caregivers need to be told that nobody else is allowed to collect the results on her behalf).
Interviewer says: Thank you very much for your time today

PARTICIPANT & FACILITY IDENTIFICATION [DO NOT SEPERATE PAGES OF THIS QUESTIONNAIRE]

EA

E01 Participant identification sticker — PLACE MOTHER STICKER HERE

E02 Date of enrolment — d d / m m m / 2 0 1 _ (y y y y)

E03 Staff ID — ☐☐☐

E04 Infant/child's birthdate — d d / m m m / 2 0 1 _ (y y y y)

E05 Sex of the child — ☐ Male ☐ Female

E06 Have you (the interviewer) reviewed the biological mother's health passport? *(if possible verify the answers with health passport records)* — ☐ Yes ☐ No

E07 How many times did you (the mother) go to ANC for this last pregnancy?
Munapita kangati ku sikelo ya a mayi oyembekezera ndi mimba ya mwana uyu? — ☐☐ *time(s)* ☐ Don't know

E08 How many months pregnant were you (the mother) at your first visit to ANC?
Kodi sikelo munayamba muli ndi mimba ya miyezi ingati? — ☐☐ *month(s)* ☐ Don't know

E09 Was the child born in a hospital or health centre?
Kodi mwanayu anabadwira kuchipatala? — ☐ No ☐ Yes ☐ Don't know

E10 Have you (the mother) ever been tested for HIV before this last pregnancy?
Kodi munayamba mwayezetsako kachilombo ka HIV musanatenge mimba ya mwana uyu? — ☐ No ☐ Yes ☐ Don't know

E11 For how many days did the baby get Nevirapine (ARV syrup)? (0-42 days)
Kodi mwana munamumwetsapo Neverapine (makhwala a ARV amadzi) kwa masiku angati? (Kuyambila tsiku 0-42) — ☐☐ *days* ☐ Don't know

E12 Have you ever received single dose Nevirapine for prevention of HIV transmission in a previous pregnancy?
Kodi mbuyomu muli ndi mimba munayamba mwalandirako Neverapine (m'bulu omwa kamodzi mimba ikawawa) omwa kamodzi poteteza kufala kwa HIV? — ☐ No ☐ Yes ☐ Don't know

E13 How many children has the biological mother given birth to?
Kodi munabeleka ana angati?
☐☐ *children*

E14 How many of the mothers' children have passed away?
Ndi angati omwe anamwalira?
☐☐ *children*
a + b + c + d

E15 How many of those deaths were: *(deaths should add up to answer given in E14)*
Mwa omwalira ndi angati amene :

a ☐☐ *stillbirths?* onabadwa akufa Kale
b ☐☐ *Infant deaths < 1 year?* anamwalira asanathe chaka chimodzi?
c ☐☐ *between 1-5 year deaths?* anamwalira pakati pa chaka chimodzi ndi zisanu?
d ☐☐ *> 5 year deaths?* anamwalira ndi zaka zoposera zisanu?

E16 Has the child had a DBS taken before today?
Kodi mwanayu anatengedwapo magazi (DBS) oti tidziwe ngati ali ndi kachilombo ka HIV Kapena ayi?
☐ No ☐ Yes ☐ Don't know

E17 If Yes, what was the DBS result?
[Ngati ndi eya] zotsatira zake zinatuluka bwanji?
☐ PCR negative ☐ PCR positive ☐ Results not received ☐ Not applicable

E18 If PCR positive, is the child on ART? *(not single dose neverapine prophylaxis)*
[Ngati zotsatira za magazi zinasonyeza kuti ali ndi kachilombo ka HIV] kodi mwanayu ali pa mankhwala a ARV?(osati omwa kamodzi a Nevirapine)
☐ No ☐ Yes ☐ Don't know ☐ Not applicable

E19 *(If not on ART)* Why is the child not on ART?
[Ngati mwana sali pa ARV], chifukwa chiyani sali pa ART?
☐ No test result yet ☐ Today was first positive result ☐ Other ☐ Not applicable

E20 If Other, specify:
Ngati pali chifukwa china fotokozani:

E21 *(If on ART)* Since the child's last clinic visit, how many times has the child missed ARVs?
(Ngati ali pa ART) kuchokera ulendo womaliza munabwera kuchipatala ndi mwanayu, kodi mwana anadumphitsa kumwa mankhwala masiku angati?
☐ Not on ART ☐ No doses missed ☐ 1 ☐ 2+

E22 What was the reason the child missed his/her ARVs? (check all that apply)
Chifukwa chiyani anadumphitsa kumwa mankhwala a ARV? (Chongani zonse zoyenera)
☐ Forgot ☐ Travel ☐ Sick ☐ Other ☐ Not applicable

E23 If Other, specify:
Ngati pali chifukwa china fotokozani:

NP06: NEMaPP ENROLMENT B QUESTIONNAIRE

print ID 5000

[TO BE ADMINISTERED IN ADDITION TO THE MAIN ENROLMENT QUESTIONNAIRE]

EB

EB01	Participant identification sticker	PLACE MOTHER STICKER HERE
EB02	What is the mother's highest level of education?<br>Kodi mayi munalekeza kalasi yanji kusukulu?	☐ none<br>☐ primary education<br>☐ secondary education<br>☐ post-secondary education
EB03	Current employment status of mother/caregiver?<br>Kodi mayi/oyang'anira mwana amachita chani?	☐ formal employment/regular job<br>☐ piece work<br>☐ own (small) business<br>☐ House wife
EB04	What is your religion?<br>Kodi mumapemphera chipembedzo chanji?	☐ Catholic<br>☐ CCAP<br>☐ Anglican<br>☐ Seventh Day Advent/Baptist<br>☐ Other Christian<br>☐ Islam<br>☐ Other<br>☐ No religion
EB05	Was this baby born at term?<br>Kodi mwanayu anabadwa okwanira masiku?	☐ no, before 38 weeks<br>☐ yes, 38-40 weeks<br>☐ after 40 weeks
EB06	What was the birth weight of your (index) baby?<br>Kodi mwanayu anabadwa ndi sikelo yanji?	☐ . ☐ *kgs -- (check the child's health passport book)*
EB07	Did you ever take antiretrovirals to prevent the transmission of HIV to your baby <u>in previous pregnancies</u>? (may check multiples)<br>Kodi munayamba mwamwako ma ARV pofuna kuteteza kupatsira HIV kwa mwana wanu pa mimba za m'mbuyomo?	☐ no, never had a previous pregnancy<br>☐ no, not taken antiretrovirals in previous pregnancies<br>☐ yes, Single dose NVP<br>☐ yes, several weeks of AZT around delivery<br>☐ yes, started on triple ART (1A)<br>☐ yes, started on triple ART (5A)<br>☐ don’t know
EB08	Were any of your previous children tested for HIV?:<br>Kodi ana ena anayezedwapo kachilombo ka HIV m'mbuyomu?	
	(a). How many children were tested negative?<br>Ndi angati sanapezeke ndi HIV?	☐☐ *children*
	(b). How many children were tested positive?<br>Ndi angati anapezeka ndi HIV?	☐☐ *children*
	(c). How many children were not tested?<br>Ndi angati sanayezedwe?	☐☐ *children* (REFER FOR TESTING)

NP06: NEMaPP ENROLMENT C QUESTIONNAIRE

[TO BE ADMINISTERED IN ADDITION TO THE MAIN ENROLMENT QUESTIONNAIRE + ENROLMENT B QUESTIONNAIRE]

Ec

EC01	Participant identification sticker	PLACE MOTHER STICKER HERE
EC02	Was this last pregnancy intended (wanted) or unwanted?<br>Kodi mimba yapitayi inali yokonzekera kapena inabwera mosakonzekera?	☐ Yes, wanted<br>☐ No, unwanted<br>☐ Don't know
EC03	Were you using contraception when you got pregnant with your (study) baby?<br>Kodi munali pa njira yolera pamene munatenga mimba ya mwana ali mu kafukufukuyu?	☐ No<br>☐ YES, Depo<br>☐ YES, Condoms always<br>☐ YES, Condoms occasionally<br>☐ YES, IUCD<br>☐ In-plants<br>☐ Oral contraceptives (the pill)<br>☐ YES, other ☐☐☐☐☐☐☐☐☐☐<br>☐ Don't know
EC04	Have you (the mother) ever been diagnosed with high blood pressure?<br>Munayamba mwapezeka ndi nthenda yothamanga magazi?	☐ No<br>☐ Yes<br>☐ Don't know
EC05	Have you (the mother) ever been diagnosed with diabetes (high blood sugar)?<br>Munayamba mwapezeka ndi matenda a sugar?	☐ No<br>☐ Yes<br>☐ Don't know
EC06	Have you ever (the mother) been treated for TB?<br>Munayamba mwalandirako mankhwala a TB?	☐ No, never<br>☐ Yes, 1 time<br>☐ Yes, more than 1 time

→ Last TB treatment start date: ☐☐ ☐☐☐ ☐☐☐☐ (d d m m m y y y y)

→ Last TB tgreatment end date: (*leave blank if still on treatment*) ☐☐ ☐☐☐ ☐☐☐☐ (d d m m m y y y y)

NP03: NEMaPP VISIT QUESTIONNAIRE

PARTICIPANT & FACILITY IDENTIFICATION [DO NOT SEPERATE PAGES OF THIS QUESTIONNAIRE]

V01 Participant identification sticker

PLACE MOTHER STICKER HERE

V A

V02 Which interview is this?
- ☐ Enrolment interview
- ☐ Follow-up Interview 1 at 12 months
- ☐ Follow-up Interview 2 at 24 months

V03 Sex of the caregiver
- ☐ Male ☐ Female

V04 Are you the biological parent of the child?
Kodi ndinu kholo lomubereka mwanayu?
- ☐ No ☐ Yes

V05 Is the mother...
Ngati siinu mayi ake, mayi ake:
- ☐ Alive
- ☐ Dead → V06

V06 Date of Mothers' death
Anamwalira liti?

d	d	m	m	m	y	y	y	y
					2	0	1	

V07 Did the mother die during child birth or within 7 days of delivery?
Kodi mayi ake anamwalira pochira kapena pasanathe masiku asanu ndi awiri atachira?
- ☐ No ☐ Not applicable
- ☐ Yes
- ☐ Unknown

V08 Cause of mother's death
Anamwalira ndi chiyani?
- ☐ Illness ☐ Not applicable
- ☐ Trauma/Accident
- ☐ Unknown

V09 How long does it take you to get to this clinic from your home?
Kodi mumatenga nthawi yaitali bwanji kuti mufike ku chipatala kuno kuchokela kwanu?
- ☐ < 1 hour
- ☐ 1-2 hours
- ☐ > 2 hours

V10 Do you have a spouse/partner?
Kodi muli ndi mwamuna?
- ☐ No
- ☐ Yes → V11
- ☐ No answer

V11 If Yes: is he the father of this last born child?
[Ngati ndi eya] Kodi ndi bambo ake wamwanayu?
- ☐ No ☐ I don't have a partner
- ☐ Yes
- ☐ No answer

V12 Does your spouse / partner know your HIV status?
Kodi mwamuna wanu akudziwapo za momwe mthupi mwanu mulili zokhuzana ndi kachilombo ka HIV?
- ☐ No ☐ I don't have a partner
- ☐ Yes
- ☐ I am not HIV positive

V13 Has your partner ever had an HIV test?
Kodi mwamuna wanuyo anayezetsapo magazi kuti adziwe ngati ali ndi kachirombo ka HIV kapena ayi?
- ☐ No ☐ I don't have a partner
- ☐ Yes (negative)
- ☐ Yes (positive)
- ☐ Don't know

V14 Are you on ART now?
Kodi muli pa mankhwala a ARV
☐ Yes (on ART now) ☐ No (never on ART) ☐ No (stopped ART)
ART #: [ ][ ][ ][ ][ ][ ][ ][ ]

V15 Have you been on another regimen before current regimen? (does not include **SdNVP)**
Kodi munayamba mwamwapo ma ARV ena musanayambe kumwa amene mukumwa panopo ?
☐ No (never before on ART) ☐ No (never other regimen) ☐ Yes (other regimen before)

V16 *(For those on ART)* How was your health when you started ART?
(Kwa amene ali pa ART) Kodi thanzi lanu linali bwanji pamene mumayamba ARV?
☐ No illness/fine ☐ A little bit sick ☐ Very sick ☐ Not applicable

V17 How do you rate your health today? Kodi thanzi lanu mukuliona bwanji lero?
☐ No illness/fine ☐ A little bit sick ☐ Very sick

V18 In the last month, how many days did you miss taking your ARVs?
Mu mwezi wapitawu, kodi mwadumphitsa kwamasiku angati osamwa mankhwala a ARV?
☐ 0 ☐ 1 day ☐ ≥2 days ☐ Not applicable

V19 What was the reason for missing your ARVs? Chifukwa chiyani munadumphitsa kumwa ma ARV?
☐ Forgot ☐ Travel ☐ Sick ☐ Other ☐ Not applicable

V20 If Other, specify:
Ngati pali chifukwa china fotokozani: [ ]

V21 Have you been diagnosed with TB in the last year?
Munayamba mwapezekapo ndi chifuwa chachikulu cha TB mu chaka chapitachi? ☐ No ☐ Yes

V22 If started TB treament, start date
[Ngati ndi eya], munali pamankhwala a TB, munayamba liti?
[d][d] [m][m][m] [y][y][y][y]

STAFF SAYS; Now I'm going to ask you some questions about the child here with you today. Please can I see the child's health passport? **Ogwira ntchito anene: Tsopano ndikufunsani mafunso okhudza mwana wanu muli naye lero. Chonde ndingaone nawo buku lakuchipatala la mwanayu?**

V23 Is the child sick or well today? Kodi mwanayu ali bwanji?
☐ Sick ☐ Well ☐ Died

V24 If died, date of death
[Ngati anamwalira], anamwalira liti?
[d][d] [m][m][m] [y][y][y][y]

V25 How many times have you brought the child to the clinic *for a health problem* in the last three months?
(check health passport to confirm). Ndi maulendo angati amene mwabwela kuchipatala ndimwanayu akudwala pa miyezi itatu yapitayi?
☐ 0 ☐ 1 ☐ 2 ☐ 3 ☐ 4 ☐ 5+

V26 Is the child already enrolled in the HIV Care Clinic *(exposed infant clinic)*?. *Kodi mwanayu analowa kale mundondomeko ya chithandizo cha ana amene ali pachiopyezo chotenga kachilombo ka HIV?*
☐ No ---- REFER! ☐ Yes ---- HCC# [ ][ ][ ][ ][ ][ ][ ][ ]

V27 Is the child taking CPT *(Bactrim)* now? Kodi mwanayu akumwa bactrim panopa? ☐ No ☐ Yes

V28 In the last seven days, what have you fed the child? *(ask each item on the list, multiple answers possible)*
Musabata yapitawa, kodi mwana mwamudyetsa chakudya chanji?
☐ Breast milk ☐ Other milk ☐ Plumpy nut ☐ Sobo/soft drink ☐ Soup ☐ Fruit ☐ Porridge
☐ Vegetables ☐ Eggs ☐ Meat ☐ Fish ☐ Nsima/rice ☐ Other

V29 Has the child ever been admitted to hospital?
Kodi mwanayu anayamba wagonekedwapo kuchipatala?
☐ No ☐ Yes, more than once ☐ Yes, 1 time ☐ Don't know

NP09: NEMaPP Visit C QUESTIONNAIRE

[TO BE ADMINISTERED IN ADDITION TO THE MAIN Visit QUESTIONNAIRE + Visit B questionnaire]

VC01 Participant identification sticker

PLACE
MOTHER STICKER HERE

Vc

VC02 Which visit is this?

☐ Enrolment interview ☐ Annual visit 1 at 12 months ☐ Annual visit 2 at 24 months ☐ Annual visit 3 at 36 months

☐ Quarterly visit 1 - Year 1 ☐ Quarterly visit 1 - Year 2 ☐ Quarterly visit 1 - Year 3 ☐ Quarterly visit 1 - Year 4

☐ Quarterly visit 2 - Year 1 ☐ Quarterly visit 2 - Year 2 ☐ Quarterly visit 2 - Year 3 ☐ Quarterly visit 2 - Year 4

☐ Quarterly visit 3 - Year 1 ☐ Quarterly visit 3 - Year 2 ☐ Quarterly visit 3 - Year 3 ☐ Quarterly visit 3 - Year 4

☐ Annual visit 4 at 48 months

Staff says: We will start with asking you some questions about the (study) child here with you today. Please can I see the child's health passport?

VC03 Since the last visit, has your child had: Kuchokera ulendo omaliza, Kodi mwana wanu wadwalako izi?

Cough/difficulty breathing: ☐ No ☐ Yes ☐ Don't know

Skin rash: ☐ No ☐ Yes ☐ Don't know

Oral thrush: ☐ No ☐ Yes ☐ Don't know

Fever: ☐ No ☐ Yes ☐ Don't know

Other: ☐ No ☐ Yes ☐☐☐☐☐☐☐☐☐☐

VC04 Rate Child's Health Status (compared to other children of the same age): (Tick One only)

Umoyo wamwana uli bwanji poyelekeza ana a msinkhu wake(chongani bokosi imodzi)

☐ Fully active, normal (100)
☐ Minor restrictions in strenuous physical activity (90)
☐ Active, but tired more quickly than other children his/her age (80)
☐ Greater restriction of play and less time spent in play activity (70)
☐ Up and around, but active play minimal; quieter activities (60)
☐ Lying around much of the day, no active playing (50)
☐ Mainly in bed; participates in quiet activities (40)
☐ Bedbound; needing assistance even for quiet play (30)
☐ Sleeping often; play entirely limited to very passive activities (20)
☐ Doesn't play; does not get out of bed (10)

VC05 Rate Mother's Health Status : (Tick One only)

zokhudza umoyo wa amayi (Chongani bokosi imodzi)

☐ Normal no complaints; no evidence of disease (100)
☐ Able to carry on normal activity; minor signs or symptoms of disease (90)
☐ Normal activity with effort; some signs or symptoms of disease (80)
☐ Cares for self; unable to carry on normal activity or to do active work (70)
☐ Requires occasional assistance, but able to care for most personal needs(60)
☐ Requires considerable assistance and frequent medical care (50)
☐ Disabled; requires special care and assistance (40)
☐ Severely disabled; hospital admission indicated/ death not imminent (30)
☐ Very sick; hospital admission necessary; supportive treatment necessary (20)
☐ Moribund; fatal processes progressing rapidly (10)

VC06	Have you (the mother) had a new diagnosis of any of the following since last visit or in the last 3 months? (tick all that applies)<br>kuchokela pamene munabwera ulendo otsiriza munayamba mwapezeka ndi izi? (chongani zonse zoyenera)	☐ Trauma ☐ Diabetes<br>☐ Malaria ☐ Tuberculosis<br>☐ Pneumonia ☐ NONE<br>☐ Diarrhea ☐ Other (specify):<br>☐ High blood pressure ☐☐☐☐☐☐☐☐☐☐
VC07	Were you ***hospitalized*** since your last study visit?<br>kuchokela pamene munabwera ulendo omaliza, mwagonekedwapo m'chipatala?	☐ No<br>☐ Yes for what? (check passport): ☐☐☐☐☐☐☐

Staff says:

I am now going to ask you some questions about symptoms you (THE MOTHER) may be feeling today or since the last study visit.
Please check all that were experienced: *1=it doesn't bother me, 2= it bothers me a little, 3=it bothers me, 4=it bothers me a lot*

					Duration	
VC08	kukhosomola	Cough	☐ No	☐ Yes	☐ <1wk ☐ 1-2 wk ☐ >2-3wk ☐ >3wk	☐ 1 ☐ 2 ☐ 3 ☐ 4
VC09	kutentha thupi	Fever	☐ No	☐ Yes	☐ <1wk ☐ 1-2 wk ☐ >2-3wk ☐ >3wk	☐ 1 ☐ 2 ☐ 3 ☐ 4
VC10	Kuchepa thupi	Weight loss	☐ No	☐ Yes	☐ <1wk ☐ 1-2 wk ☐ >2-3wk ☐ >3wk	☐ 1 ☐ 2 ☐ 3 ☐ 4
VC11	Kutuluka thukuta usiku	Night sweats	☐ No	☐ Yes	☐ <1wk ☐ 1-2 wk ☐ >2-3wk ☐ >3wk	☐ 1 ☐ 2 ☐ 3 ☐ 4

IF YES, TO ONE OR MORE SYMPTOMS ABOVE (VC08-VC11), REFER FOR SPUTUM SCREENING

Please ensure patient is flagged in register (cough register) to track sputum result.

VC12 Did you submit sputum since the last visit? ☐ No ☐ Yes

VC13	Phuma	Shortness of breath	☐ No	☐ Yes	☐ <1wk ☐ 1-2 wk ☐ >2-3wk ☐ >3wk	☐ 1 ☐ 2 ☐ 3 ☐ 4
VC14	Mseru kapena kusanza	Nausea and/or vomiting	☐ No	☐ Yes	☐ <1wk ☐ 1-2 wk ☐ >2-3wk ☐ >3wk	☐ 1 ☐ 2 ☐ 3 ☐ 4
VC15	Kutsegula mmimba	Diarrhea or loose bowel movements	☐ No	☐ Yes	☐ <1wk ☐ 1-2 wk ☐ >2-3wk ☐ >3wk	☐ 1 ☐ 2 ☐ 3 ☐ 4
VC16	Mutu kupweteka	Headache	☐ No	☐ Yes	☐ <1wk ☐ 1-2 wk ☐ >2-3wk ☐ >3wk	☐ 1 ☐ 2 ☐ 3 ☐ 4
VC17	ululu, dzanzi kapena kubayabaya kwa mapazi ndi manja	Pain, numbness or tingling in the hands or feet	☐ No	☐ Yes	☐ <1wk ☐ 1-2 wk ☐ >2-3wk ☐ >3wk	☐ 1 ☐ 2 ☐ 3 ☐ 4
VC18	Zilonda zotuluka mthupi	Rash	☐ No	☐ Yes	☐ <1wk ☐ 1-2 wk ☐ >2-3wk ☐ >3wk	☐ 1 ☐ 2 ☐ 3 ☐ 4
VC19	Kuvutika kukumbukira/kubalalika	Trouble remembering or confusion	☐ No	☐ Yes	☐ <1wk ☐ 1-2 wk ☐ >2-3wk ☐ >3wk	☐ 1 ☐ 2 ☐ 3 ☐ 4
VC20	kudandaula/ kukhumudwa	Feeling sad, down or depressed	☐ No	☐ Yes	☐ <1wk ☐ 1-2 wk ☐ >2-3wk ☐ >3wk	☐ 1 ☐ 2 ☐ 3 ☐ 4
VC21	nkhawa	Feeling nervous or anxious	☐ No	☐ Yes	☐ <1wk ☐ 1-2 wk ☐ >2-3wk ☐ >3wk	☐ 1 ☐ 2 ☐ 3 ☐ 4
VC22	Kulephera/Kuvutika kugona	Difficulty falling or staying asleep	☐ No	☐ Yes	☐ <1wk ☐ 1-2 wk ☐ >2-3wk ☐ >3wk	☐ 1 ☐ 2 ☐ 3 ☐ 4
VC23	Kusowa chilakolako cha chakudya	Loss of appetite	☐ No	☐ Yes	☐ <1wk ☐ 1-2 wk ☐ >2-3wk ☐ >3wk	☐ 1 ☐ 2 ☐ 3 ☐ 4

VC24	Muscle aches or joint pain<br>Kuphwanya kwa thupi	☐ No ☐ Yes	☐ <1wk ☐ 1-2 wk<br>☐ >2-3wk ☐ >3wk	☐ 1 ☐ 2 ☐ 3 ☐ 4
VC25	Yellow eyes<br>m'maso mwa chikasu	☐ No ☐ Yes	☐ <1wk ☐ 1-2 wk<br>☐ >2-3wk ☐ >3wk	☐ 1 ☐ 2 ☐ 3 ☐ 4
VC26	General fatigue or weakness<br>Kumva kutopa kapena kufooka	☐ No ☐ Yes	☐ <1wk ☐ 1-2 wk<br>☐ >2-3wk ☐ >3wk	☐ 1 ☐ 2 ☐ 3 ☐ 4
VC27	Abnormal or disturbing dreams<br>Kulota maloto oopsya/odabwitsa	☐ No ☐ Yes	☐ <1wk ☐ 1-2 wk<br>☐ >2-3wk ☐ >3wk	☐ 1 ☐ 2 ☐ 3 ☐ 4
VC28	Hearing voices or Seeing things that other people don't see (ie. visual or auditory hallucinations) Kumva mau omwe anthu ena sakumva kapena kuwona zinthu zomwe anthu ena sakuona.	☐ No ☐ Yes	☐ <1wk ☐ 1-2 wk<br>☐ >2-3wk ☐ >3wk	☐ 1 ☐ 2 ☐ 3 ☐ 4
VC29	Suicidal thoughts or attempts<br>Malingaliro ofuna kudzipha	☐ No ☐ Yes	☐ <1wk ☐ 1-2 wk<br>☐ >2-3wk ☐ >3wk	☐ 1 ☐ 2 ☐ 3 ☐ 4
VC30	Bizarre, inappropriate or aggressive behavior (not in keeping with usual conduct)<br>Makhalidwe odabwitsa/osakhala bwino/andewu	☐ No ☐ Yes	☐ <1wk ☐ 1-2 wk<br>☐ >2-3wk ☐ >3wk	☐ 1 ☐ 2 ☐ 3 ☐ 4
VC31	Strange, overvalued ideas eg about religion, being in danger, being important (delusions)<br>Kukhala ndi maganizo achilendo/odabwitsa ngati okhudza chipembedzo, kapena kukhala pachiwopsyezo, kapena kukhala ofunikira.	☐ No ☐ Yes	☐ <1wk ☐ 1-2 wk<br>☐ >2-3wk ☐ >3wk	☐ 1 ☐ 2 ☐ 3 ☐ 4

VC32 Do you drink alcohol?
Kodi mumamwa mowa?
☐ No ☐ Yes, Occasionally ☐ Yes, Every day

VC33 What kind of contraceptives are you using now? (Check all that apply)
Kodi mukugwiritsa njira yanji ya kulera?
☐ None ☐ DEPO ☐ Condoms, always ☐ Condoms, sometimes ☐ IUCD ☐ Other [ ] ☐ In-plants ☐ Oral contraceptives (the pill)

VC34 Would you like to have another baby?
Kodi mukufuna mwana wina?
☐ Yes, now or soon (<=12months) ☐ Yes, but in the future (>12 months) ☐ No ☐ Don't know

VC35 Are you currently pregnant?
Kodi ndinu oyembekezera?
☐ No ☐ Don't know ☐ Yes [ ] month(s)

PLEASE TAKE BLOOD PRESSURE

VC36 Blood pressure (mmHg): [ ][ ][ ] / [ ][ ][ ]
Systolic Diastolic

Staff says: **FOR ANNUAL VISITS ONLY**
LIPODYSTROPHY SCREEN: We are now going to ask some questions regarding changes to your body that may be occurring from HIV and ART. PLEASE PROCEED TO THE LIPODYSTROPHY QUESTIONNAIRE.
We would now like to take a blood and urine sample from the mother to test on site (protein)
This test will give us more information about your health.

VC37 Point-Of-Care TESTS
urine protein test ☐ Negative ☐ Trace ☐ + ☐ ++ ☐ +++ ☐ ++++
